# Overall Evaluation of Antibiotics Occurrence from Large-Scale Livestock Farms in Sichuan Basin, China: Spatial Distribution, Source Apportionment, and Risk Assessment

**DOI:** 10.3390/toxics13030154

**Published:** 2025-02-23

**Authors:** Changmiao Lai, Zhikai Wang, Teng Gu, Lei Jian, Xiaoxia Meng, Qingjie Meng, Dongdong Gao

**Affiliations:** 1 Sichuan Academy of Eco-Environmental Sciences, Chengdu 610041, China; yiyi06072025@163.com (C.L.); kylewang0103@126.com (Z.W.); guteng0913@gmail.com (T.G.); jianlei327@hotmail.com (L.J.); scshky@yeah.net (X.M.); 2 Sichuan Province Ecological Environment Monitoring Station, Chengdu 610031, China; mengqingjie202501@163.com

**Keywords:** antibiotics, livestock manure, source apportionment, risk assessment, Sichuan Basin

## Abstract

The widespread application of antibiotics in intensive livestock production is increasingly contributing to antibiotic contamination, and their potential ecological risk to environmental media by resourceful utilization of livestock manure as fertilizers in China has been recognized. This study conducted a comprehensive investigation on 79 large-scale livestock farms and collected 86 livestock excrements and 20 soil and 20 surface water samples distributed in Sichuan Basin, where no similar studies were carried out before. In total, four tetracyclines (TCs), eight sulfonamides (SAs), and eight fluoroquinolones (QNs) were monitored by liquid chromatography–triple quadrupole mass spectrometry. The findings revealed that antibiotics occurrence varied remarkably in excrement (feces or manure) among different livestock farms and different livestock species, following the descending order as QNs > TCs > SAs of detection rates and as TCs > QNs > SAs of detected concentrations, respectively. By source apportionment, livestock manure was demonstrated as a possible source for TCs and QNs detected in soil, while the detection of antibiotics in surface water was probably related to other sources. The central, south, and southwest of Sichuan Basin displayed a higher contamination of antibiotics from livestock manure. The ecological risk of antibiotics was obtained from a medium to heavy level, particularly TCs from swine farms to green algae, water flea, and inflated duckweed in aquatic water and QNs from all livestock farms to sensitive organisms in soil. Overall, the prioritized resource utilization of livestock manure would probably increase the contamination level and ecological risk to environment; hence, rational and effective measurement was highly recommended for antibiotics prevention in some regions of Sichuan Basin.

## 1. Introduction

Antibiotics, as one of the typical emerging pollutants, have gradually aroused a global concern in the last decade for their potential adverse impact on microorganisms, eco-environment, and human health [1,2]. Multiple sources are responsible for the extensive presence of antibiotic residues; however, veterinary sources account for more than 52% of the total antibiotic contamination when comparing with human medical treatment and municipal sewage disposal [3,4,5]. For instance, veterinary antibiotics were confirmed the main source of antibiotic residues in the lower-middle reaches of the Yangtze River [6] and in coastal water of Beibu Gulf [7]. Admittedly, antibiotics are widely used to prevent disease and to promote the growth of animals. According to a global estimation, the stable growth trend of veterinary antibiotics consumption will continue until 2030, reaching to a total volume of 105,596 ± 3605 tons [8]. As an important destination for the consumption of veterinary antibiotics, livestock and poultry breeding (LPB) is undoubtedly responsible for the large amount of antibiotics discharge, which needs deeply investigated.

LPB in China has been undergoing a scaling-up transformation from a traditional household production to an intensive industrial production [9], with an estimated 100,800 tons of veterinary antibiotics consumption every year [1]. Unfortunately, most antibiotics are difficult to be directly absorbed by the gastrointestinal tract of animals, and approximately 40–90% of them are discharged in their original form or as metabolites in livestock urine and feces [10,11]. By the routine application of livestock manure as fertilizers, antibiotics are ultimately released into environmental media through runoff, leachate, or permeation [12,13,14], leading to toxicity, persistence, and bioaccumulation, as well as the development and spread of antibiotic resistance genes (ARG) [15,16,17,18] and arousing considerable challenge on the environment protection and preservation at the same time. Therefore, it is essential to comprehensively understand the occurrence and evaluate the risk of antibiotics in different livestock excrement, to guide manure application and management for optimal antibiotics prevention.

Generally, quite a lot of veterinary antibiotics are adopted in LPB globally. The three most persistent classes of antibiotics detected in livestock excrement were sulfonamides (SAs), fluoroquinolones (QNs), and tetracyclines (TCs) [19]. Their concentrations vary significantly by antibiotic class, livestock species, geographical origin, and the type of livestock farms, in the range of a few micrograms to hundreds of milligrams per kilogram [20]. For instance, SAs in poultry manure obtained a higher concentration as 89 mg·kg^−1^ in the U.S., as compared to China with 15.93 mg·kg^−1^, while TCs in swine manure were opposite as 37 mg·kg^−1^ in the U.S. and 354 mg·kg^−1^ in China [21]. In Europe, SAs (e.g., sulfadiazine) dominated in poultry manure with a higher concentration as 10.1 mg·kg^−1^ in Albania and Kosovo, and TCs were more common in cattle manure [22]. As for swine manure, nine antibiotics were detected with a maximum concentration of 764.4 mg·kg^−1^ in Shandong Province, China [19], whereas Zhu et al. identified 14 different antibiotics in 36 Chinese swine farms at concentrations of 0 to 15.2 mg·kg^−1^ [23]. Although some studies have reported that only low levels of antibiotics remained in manure-based fertilizers, their original contamination in livestock feces or manure have still remained unclear in some regions of China, as well as their related adverse environmental impact.

To date, quite a few Chinese researchers have focused and investigated the original source of antibiotics from livestock excrement in some provinces, including Ningxia [24], Jiangxi [25], Liaoning [26], Shanghai [27], Zhejiang [28], Jiangsu [19], Tianjin [29] and Shandong [30], etc. According to above-mentioned literature, TCs, SAs, and QNs were usually obtained a relatively higher level in livestock excrement, which increasing the potential ecological risk to soil or surface water in various degrees. However, as an important LPB industry province in China, no similar reports have been identified for Sichuan yet, where its economy is underdeveloped. Considering the differences in natural geographical regions and the uneven distribution of livestock breeding scale, the occurrence and its associated influence of antibiotics probably exit significant distinction. Hence, it will be of great interest to provide more information about the sources of antibiotics in livestock manure and the surrounding environment media.

In summary, this study aims to give an exhaustive report on the spatial distribution, source apportionment and ecological risk assessment of 20 antibiotics coming from large-scale livestock farms in Sichuan Basin. The specific goals are as follows: (a) to reveal antibiotics occurrence in soil, surface water, and livestock excrement from different livestock farms and species; (b) to display spatial distribution of antibiotics contamination from different livestock farms by resourceful utilization of livestock manure; (c) to identify potential relationship of antibiotics occurrence between livestock manure and environmental media; (d) to evaluate ecological risks of antibiotics posed by the encouraged utilization of livestock manure as fertilizer.

## 2. Materials and Methods

### 2.1. Study Area and Sample Collection

**Figure 1:**
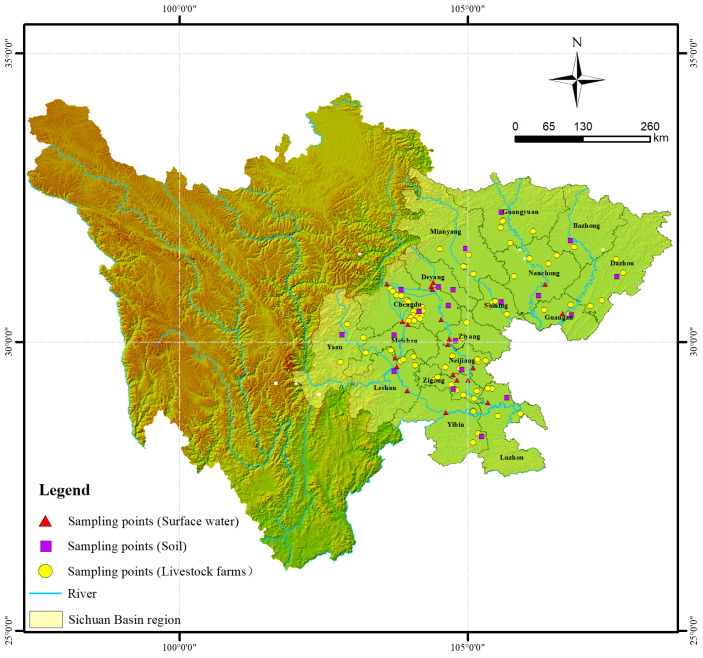
Locations of study area and sampling points distribution.

Sichuan Basin, a total area of approximately 260,000 km^2^, is mainly located in the central and eastern part of Sichuan Province, covering 17 out of 21 prefecture-level municipal units (Figure 1), along with relatively developed economy and concentrated population distribution. LPB industry is highly developed in this region, meeting the demand for meat both inside and outside. According to statistical data from Sichuan Provincial Bureau of Statistics in 2022, LPB scales in these 17 municipal units are 37,410,260 swine, 3,169,610 cattle, 8,702,241 sheep and 433,951,911 layers, respectively, accounting for approximately 70% of the total LPB scale (all converted to swine scale) in whole Sichuan (Table S1). Therefore, the study area is characterized by representativeness and typicality.

In the whole study area, a total of 79 livestock farms were selected and distributed across 17 municipal units in Sichuan Basin, including 64 swine farms, 6 layer farms, 6 cattle farms, and 3 sheep farms, respectively (Figure 1). From October in 2022 to April in 2023, 20 soil samples, 20 surface water samples, and 86 excrement samples (53 solid forms and 33 liquid forms) were collected by different batches. The locations of sample collection were as follows: all feces samples were collected from manure pits, whereas solid manure samples originated from anaerobic fermentation facilities (swine) and heap piles (cattle, layer, and sheep). Swine liquid manure was obtained from oxidation ponds or ecological ponds used for swine wastewater storage. In addition, all samples were transported to the laboratory within 24 h after collection using refrigerating equipment under 4 °C. Surface water and liquid form samples were analyzed within 48 h, while soil and solid form samples were refrigerated at −20 °C before analysis.

### 2.2. Target Antibiotics

Totally 20 antibiotics, including 4 TCs, 8 SAs, and 8 QNs were selected and analyzed in this study, which covered the commonly used on livestock farms and frequently detected in livestock excrement and environment [19,31,32]. The full names, acronyms and physicochemical properties of antibiotics are shown in Table S2. All target antibiotics were purchased from Sigma-Aldrich (Shanghai, China). The purity of all standards was above 98%.

### 2.3. Sample Pretreatment

The pretreatment for liquid form samples was as follows: 500 mL samples were filtered by 0.7 μm glass fiber membrane. Hydrochloric acid was added to the filtered samples to reach pH around 3.0. Solid phase extraction (SPE) was used by CNW Poly-Sery HLB Pro column. The column was activated sequentially with 5 mL methanol, 5 mL deionized water and 5 mL hydrochloric acid solution with pH = 2.0, then the samples were passed through at a flow rate of 10 mL·min^−1^. Next, the extraction column was rinsed by 5 mL deionized water, and blown to nearly dry by nitrogen, followed by eluting with 5 mL methanol and 5 mL methanol–acetonitrile mixture (*v*:*v* = 1:1). The eluent was finally fixed to a volume of 1 mL with methanol after blowing with nitrogen to nearly dryness, filtered by using 0.22 μm nylon syringe filters, and stored for further analysis.

The pretreatment for the solid samples was as follows: the samples were frozen to dry firstly and then grinded and passed through a 0.25 μm pore size sieve. Next, 1.0 g samples were weighed and added to 50 mL centrifuge tubes. Each tube received 10 mL acetonitrile–McIlvaine buffer and 0.5 g EDTA, followed by vortex mixing. Then, all tubes were ultrasonicated at 80 kHz for 15 min with temperature of 35 °C and centrifuged at 10,000 rpm for 10 min. In addition, 5 mL methanol was added to the residue, followed by vortex mixing, ultrasonication, and centrifugation sequentially. The extraction was repeated a second time following the same steps as above. Both the methanol phase and the acetonitrile phase supernatants were mixed in a new tube, then diluted to 500 mL with ultrapure water. After adjusting pH to around 3.0, the samples were purified and enriched by SPE using CNW Poly-Sery HLB Pro column according to the aqueous pretreatment process.

### 2.4. Instrumental Analysis

Liquid chromatography–triple quadrupole mass spectrometry (AB SCIEX 3200 Q TRAP LC-MS/MS, USA) was used to analysis antibiotics in samples. The ultra-high performance liquid chromatography BEH C18 1.7 μm column (100 mm × 2.1 mm) was used for separation. The column oven temperature was maintained at 40 °C. The mobile phase A was ultrapure water containing 0.1% (*v*/*v*) formic acid and 2.0 mmol·L^−1^ ammonium acetate, and phase B was chromatographically pure methanol. The flow rate was 0.2 mL·min^−1^ with a gradient elution program as follows: 0–5 min, 90–85% B; 5–7 min, 85–80% B; 7–10 min, 80–60% B; 10–12 min, 60–40% B; 12–14 min, 40–25% B; 14–16 min, 25–10% B; 16–18 min, 10% B; 18–20 min, 10–90% B; 20–21 min, 90% B. The injection volume was 10 μL.

For all antibiotics, the MS instrument was operated in the electrospray ionization (ESI) positive mode and the data were acquired in the multiple reaction monitoring (MRM) mode. The capillary voltage was 3.0 kV, the source temperature was 150 °C, and the desolvation gas temperature was 500 °C. The cation scanning mode was used to optimize the parameters related to MS/MS analysis. Optimization results are all detailed in Table S3.

### 2.5. Quality Assurance and Control (QA/QC)

Stringent quality control procedures were performed throughout the pretreatment and analysis. Quantification of target antibiotics was accomplished by internal indicators, and the relative standard deviation (RSD) was normally below 15%. The standard curve concentration ranges were from 0.01 to 500 μg·L^−1^, with linear correlation coefficients above 0.99 (Table S4). The detection limits of liquid sample were 0.0001, 0.0001, and 0.002 ng·L^−1^ for TCs, SAs, and QNs, respectively, whereas the detection limits of solid sample were 0.3, 0.2, and 0.3 μg·kg^−1^, respectively.

### 2.6. Source Apportionment Methods

Principal components analysis (PCA) was adopted to analyze the characteristics of antibiotics in soil, surface water and manure samples. To select the corresponding principal factors, their eigenvalues should be above 1 after normalization method [33]. Data analysis and processing were performed using Origin 2017 software.

### 2.7. Potential Ecological Risk Assessment of Antibiotics

The risk quotient (RQ) approach was usually used to evaluate the potential ecological risk of antibiotics to ecosystems [19,29,33]. The RQ values were divided into four levels of risk: (a) insignificant risk (<0.01), (b) low risk (0.01–0.1), (c) medium risk (0.1–1), and (d) high risk (>1). The RQ calculation formula is as follows:

(1) $$\mathrm{RQ}=\frac{\mathrm{MEC}}{{\mathrm{PNEC}}_{water/soil}}$$

where *MEC* represents the measured environmental concentration of antibiotic and *PNEC_water/soil_* represents the predicted no-effect concentration of antibiotic on aquatic or terrestrial creature.

Based on classical ecotoxicological tests, species sensitivity distributions (SSD) was recommended to derive specific *PNEC_water_* of antibiotics in this study [34], which estimating the hazardous concentration (HC_5_) corresponding to a certain proportion (usually under 5%) of numerous EC_50_ or LC_50_ values of individual species when they are adversely affected [35]. In addition, according to the Technical Guidance Document (TGD) on Risk Assessment [36], the *PNEC_soil_* of antibiotics could be calculated through formulas from (2) to (6).

(2) $${\mathrm{PNEC}}_{\mathrm{water}}=\frac{{\mathrm{HC}}_{5}}{\mathrm{AF}}$$

(3) $${\mathrm{PNEC}}_{\mathrm{soil}}=\frac{K_{soil\text{-}water}}{{\mathrm{RHO}}_{\mathrm{solid}}}\times {\mathrm{PNEC}}_{\mathrm{water}}\times 1000$$

(4) $$K_{soil\text{-}water}=F_{water\text{-}soil}+F_{solid\text{-}soil}\times \frac{{\mathrm{Kp}}_{\mathrm{soil}}}{1000}\times {\mathrm{RHO}}_{\mathrm{solid}}$$

(5) $${\mathrm{Kp}}_{\mathrm{soil}}={\mathrm{Foc}}_{\mathrm{soil}}\times K_{\mathrm{oc}}$$

(6) $$\log K_{\mathrm{oc}}=0.623\;\times \log K_{\mathrm{ow}}+0.873$$

where *AF* represents the assessment factor (this study takes the value as 1); *K_soil-water_* represents the soil–water partition coefficient, m^3^·m^−3^; *RHO_solid_* represents solid density (the value defined in TGD is 2500 kg·m^−3^); *F_water-soil_* and *F_solid-soil_* represent the volume fractions of the water and solid phases in the soil, respectively (the values defined in TGD are 0.2 and 0.6 m^3^·m^−3^, respectively); *Kp_soil_* represents the solid phase–water partition coefficient, L·kg^−1^; *Foc_soil_* represents the mass fraction of organic carbon in soil solids (the value defined in TGD is 0.02 kg·kg^−1^); *K_oc_* represents the organic carbon–water partitioning coefficient; *K_ow_* represents the octanol–water partition coefficient.

The *PNEC_water_* and *PNEC_soil_* values of antibiotics for different species are provided in Table 1. All EC_50_ or LC_50_ data from classical ecotoxicological tests in this study were obtained from Environmental Protection Agency (EPA) ECOTOX database (https://cfpub.epa.gov/ecotox/, accessed on 4 November 2024).

## 3. Results and Discussion

### 3.1. Antibiotics Occurrence Among Different Livestock Excrement

**Figure 2:**
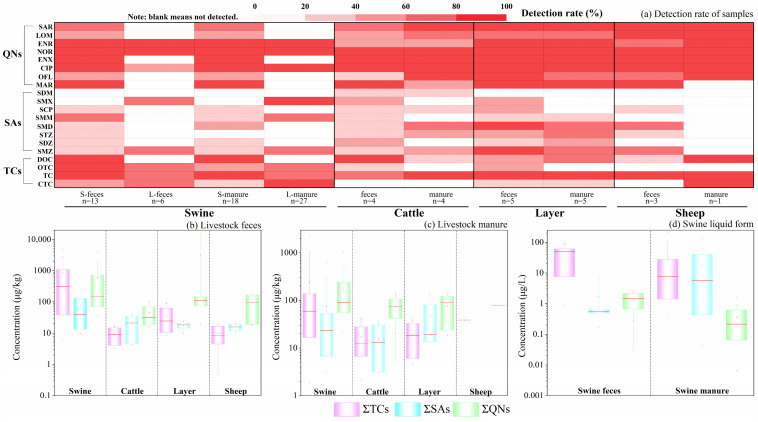
Detection rates (**a**) and detected concentrations of antibiotics in different livestock feces (**b**), manure (**c**), and in swine wastewater (**d**), respectively. (A deeper red color corresponds to a higher detection rate.).

As clearly shown in Figure 2a, except swine wastewater, the overall detection rates of all the investigated antibiotics among different livestock species, both in feces samples (Table S5) and manure samples (Table S6), basically followed the descending order as QNs > TCs > SAs. Specially, all eight QNs, three TCs (DOC, OTC and TC), and two SAs (SMD and SMZ) were significantly detected with detection rates almost above 80%, 60%, and 40%, respectively. Compared with different livestock species, the detection rates and detected types of antibiotics in layer farms were the largest, followed by those in swine farms and cattle farms. These results demonstrated the high prevalence of antibiotic contamination in livestock farms, and the intensity in layer and swine farms was probably more severe.

Figure 2b,c show the concentration and its distribution of antibiotics in feces and manure samples from different livestock farms. Obviously, different livestock species exhibited remarkably varying concentrations of antibiotics in their excrement. In swine farms, the descending orders of average concentrations in feces and manure were TCs (863 and 235 μg·kg^−1^) > QNs (593 and 181 μg·kg^−1^) > SAs (75 and 74 μg·kg^−1^). This result was different from Jiangsu Province, where the descending order of concentrations in swine farms was SAs (69.2 μg·kg^−1^) > TCs (19.34 μg·kg^−1^) > QNs (3.72 μg·kg^−1^) [19]. In layer and cattle farms, QNs obtained the highest concentrations as 3155 ± 6858 μg·kg^−1^ and 45 ± 38 μg·kg^−1^ in feces and 76 ± 51 μg·kg^−1^ and 75 ± 46 μg·kg^−1^ in manure, respectively. Meanwhile, the concentration of TCs in cattle farms was slightly higher than that of SAs, where in layer farms, SAs were higher than TCs, indicating that the breeding dosage of antibiotics existed distinction according to different livestock growth habits, farming methods, etc. Additionally, it was worth noting that the varied concentration of antibiotics still existed among different livestock farms even raising same species. For instance, the minimum and maximum values of QNs in layer feces samples were 19 and 15,424 μg·kg^−1^, respectively, with a difference of approximately 800 times; while TCs in swine manure samples reached a maximum concentration of 2284 μg·kg^−1^, which is four orders of magnitude higher than the minimum concentration of 2 μg·kg^−1^. All these detected concentrations of each single antibiotic were lower than previous study as 15,200 μg·kg^−1^ (maximum value) in other region of China [23], indicating the utilization of veterinary antibiotics in Sichuan was under optimized control comparatively.

As shown in Figure 2a,d, just 9 out of 20 target antibiotics were detected in swine wastewater, including three TCs (OTC, TC, and CTC), three QNs (ENR, NOR, and CIP) and three SAs (SMX, SMM, and SMZ), with detection rates ranging from 20% to 100%. Moreover, the descending orders of average concentrations in feces and manure were TCs (40.9 and 27.5 μg·L^−1^) > SAs (1.7 and 19.1 μg·L^−1^) > QNs (1.4 and 0.4 μg·L^−1^), demonstrating most antibiotics, like TCs and QNs, were inclined to remain to solid particles [10]. In fact, OTC and CTC have been widely used in the prophylaxis of bovine pneumonia and diarrhea in calves and piglets [20], relating to their relatively higher detection rates and concentrations. However, the concentration of SAs increased in swine wastewater, probably due to their positive logK_ow_ values of SMX, SMM, and SMZ (Table 1), causing them more easily soluble in water [37]. Zhi et al. also found that SMX and SMM were dominated in swine wastewater [38].

All above findings suggested that (a) high-prevalence utilization of TCs, QNs, and SAs in livestock farms probably leads to antibiotics contamination to the environment by fertilizing livestock manure unreasonably; (b) TCs and QNs were in a higher quantity than SAs in livestock excrement, especially swine and layer, consistent with previous findings [39]. (c) Antibiotic concentrations of livestock manure were probably affected by different treatment process adopted in livestock farms.

### 3.2. Antibiotics Occurrence in Soil and Surface Water

As listed in Table 2, 11 out of 20 antibiotics were detected in soil samples, including 4 TCs and 7 QNs, with total concentrations ranging from N.D to 7.8 μg·kg^−1^ of TCs and from 13.9 to 29.8 μg·kg^−1^ of QNs, respectively. Among QNs, CIP and NOR had the highest detection rates (100%), followed by ENX (95%), MAR (90%), and LOM (65%). While among TCs, just TC obtained a detection rate above 60%, illustrating QNs were the most abundant contaminants in soil, which was similar to a previous study in Jiangsu [33]. Actually, TCs and QNs intended to persist for several months to years in soil [40]. The detected antibiotics concentrations were in the range of N.D. to 12.2 μg·kg^−1^, remarkably lower than some regions in China, where TCs were reported to be as high as 1590 μg·kg^−1^ [41], suggesting the level of antibiotic contamination in soil remained within an acceptable range in Sichuan Basin.

As listed in Table 3, 16 out of 20 antibiotics were detected in surface water samples, including 4 TCs, 6 SAs, and 6 QNs, with total concentrations ranging from N.D to 0.008 ng·L^−1^ of TCs, from 0.00152 to 0.1845 ng·L^−1^ of SAs and from 0.00478 to 0.5067 ng·L^−1^ of QNs, respectively, mainly remaining under ng·L^−1^ level. This result is lower than the previous findings, which suggesting antibiotics concentrations typically range from ng·L^−1^ to μg·L^−1^ in various natural waters [32,42]. For instance, the total mean concentration of four TCs detected in Chishui river (Guizhou Province) was 8.38 ng·L^−1^ [43], much higher than that in Sichuan Basin, which was probably related to the administrative regulations of Sichuan Province prohibiting the direct discharge of livestock wastewater into water bodies.

Interestingly, none of the SAs were detected in soil, while most SAs were detected in surface water with higher detection rates than TCs and QNs, where SMZ and SDZ obtained the highest detection rate as 100%, followed by SCP as 90%. According to previous studies, SAs were poor chelates and more easily biodegraded [37], which possibly led to their frequent detection in water, but they were rarely detected in soils as a consequence. In contrast, TCs and QNs were more easily adsorbed to soil [5]. Overall, the occurrence of target antibiotics in environmental media (soil or water) was characterized by large variety with low concentration.

### 3.3. Source Apportionment of Antibiotics in Soil and Surface Water

**Figure 3:**
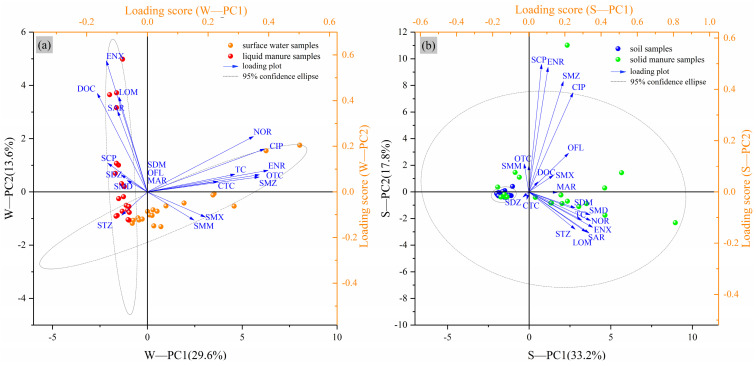
Principal component analysis (PCA) of the target antibiotics. (**a**) Surface water and liquid manure and (**b**) soil and solid manure.

To identify possible antibiotics sources, surface water and soil were compared with liquid and solid manure, respectively, by using PCA. According to Figure 3a, antibiotics like ENR, NOR, CIP, SMZ, and OTC showed higher positive loadings (>0.3) on W-PC1, which explained 29.6% of the total variance with an eigenvalue of 5.04. Meanwhile, ENX, LOM, SAS, and DOC showed higher positive loadings (>0.3) on W-PC2, which explained 13.6% of the total variance with an eigenvalue of 2.32. However, according to the distribution of principal component scores, it was inferred that antibiotics in surface water had slightly correlation with liquid manure, since its ellipse intersected partially with the ellipse of liquid manure. Besides livestock source, many anthropogenic sources could be responsible for the occurrence of antibiotics detected in surface water. Li et al. detected 13 antibiotics (OTC, TC, CIP, ENR, and NOR) in the outlet water from three municipal wastewater treatment plants located in Chengdu (central of Sichuan Basin) [44], while in a hospital located in Mianyang (north of Sichuan Basin), SAs were detected as high as 520 ng·L^−1^ in its wastewater. Additionally, pharmaceutical wastewater without proper treatment and uncontrolled release of aquaculture effluent were also important drivers of antibiotic contamination in surface waters [45]. Therefore, antibiotics in surface water would probably originate from human medical treatment, aquaculture effluent, or municipal sewage disposal [5,46], rather than discharges of swine wastewater in Sichuan Basin, which was strictly prohibited by the Government.

In contrast, as shown in Figure 3b, antibiotics in soil could probably inter-relate with livestock solid manure, since its ellipse was entirely inside the ellipse of solid manure. The eigenvalues of S-PC1 and S-PC2 were 7.60 and 3.20, explaining 38.0% and 16.0% of the total variance, respectively. Three SAs (SCP, SMZ, and SMD), six QNs (SAR, ENR, ENX, CIP, NOR, and LOM) were the dominated loading components (positive loadings > 0.3), which were not restricted in animal husbandry [47] and detected in swine and layer solid manure samples (Table S6), indicating that livestock manure would be a possible and key source of antibiotics residue in soil by fertilizing.

### 3.4. Spatial Distribution of Antibiotic from Livestock Farming in Sichuan Basin

**Figure 4:**
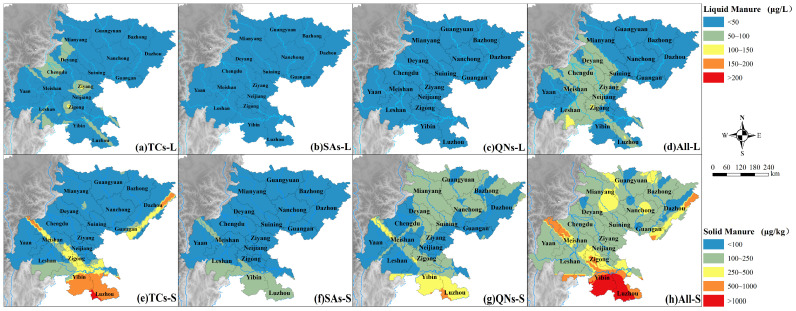
The spatial distribution of antibiotics from livestock farming in Sichuan Basin. (**a**) TCs in liquid manure; (**b**) SAs in liquid manure; (**c**) QNs in liquid manure; (**d**) all investigated antibiotics in liquid manure; (**e**) TCs in solid manure; (**f**) SAs in solid manure; (**g**) QNs in solid manure; and (**h**) all investigated antibiotics in solid manure, respectively.

Usually, the contamination level of antibiotics could be judged by concentration [48]. Considering the varied content of antibiotics in liquid or solid manure from different livestock farms, it is necessary to reveal the spatial distribution of antibiotics, which was executed through the ArcGIS platform (v10.8), in different regions of Sichuan Basin for prevention of antibiotics precisely and accurately. As shown in Figure 4, distribution of antibiotics from livestock manure displays distinct spatial variations. For liquid manure, TCs obtained a higher contamination in some regions of Chengdu, Ziyang, Zigong, Leshan, Deyang, and Mianyang. However, after adding SAs and QNs, the scope of higher contamination had expanded to almost entire central and partial southern regions of Sichuan Basin, possibly increasing the contamination extent in surface water around Minjiang and Tuojiang River basin. While for solid manure, livestock farms located in Yibin and Luzhou had a higher contamination of TCs, SAs, and QNs compared with other regions. As a consequence, a heavy contamination of antibiotics from livestock manure was undoubtably concentrated in the south of the Sichuan Basin. Meanwhile, western Chengdu, northern Yaan, southern Leshan, and eastern Dazhou also obtained a higher emission of TCs and QNs, which was mainly related to swine and layer production in the regions [49]. In summary, antibiotics from solid manure exhibited greater pollution level and larger impact range than those from liquid manure, along with the development of intensive and large-scale livestock husbandry [50], for environmental concern, stringent regulation or management should be implemented for rational use control of antibiotics, scientific excrement treatment, and reasonable manure fertilizing, furthermore, much more attention should be paid in livestock farms located in southwest of Sichuan Basin, including Yibin, Luzhou, and Zigong.

### 3.5. Ecological Risk Assessment of Antibiotics in Soil, Surface Water and Livestock Manure

Exposure to antibiotics, even at low concentrations, may cause hazardous impact on different organisms in their early life stage, leading to potential ecological risks consequently. In order to avoid the adverse effect of antibiotics on species to the greatest extent, this study calculated RQ by using the HC_5_ values of different species according to the SSD method (Table 1), resulting in a higher risk assessment intentionally. To more accurately assess the ecological risks of antibiotics in livestock manure across different farming locations, the calculation of RQ utilized the actual measured value from each individual sample rather than relying on average or maximum concentration values.

**Figure 5:**
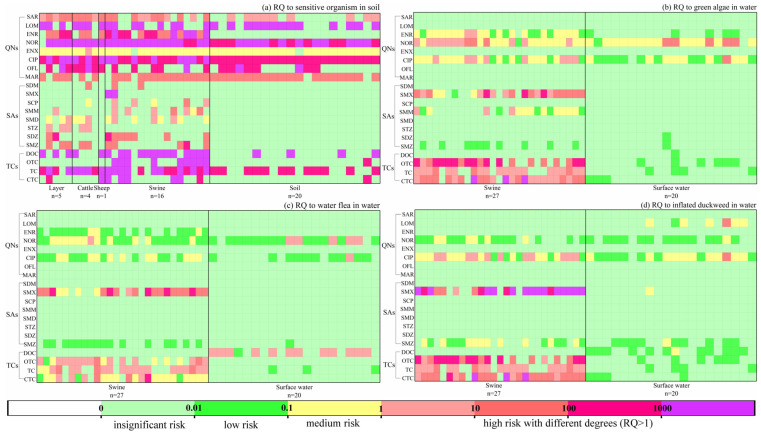
Ecological risk assessment of antibiotics in soil, surface water and livestock manure. (**a**) to sensitive organism in soil; (**b**) to green algae in water; (**c**) to water flea in water and (**d**) to inflated duckweed in water, respectively.

Figure 5a shows the ecological risk assessment of antibiotics to sensitive organisms in soil. Overall, all investigated antibiotics posed a medium or high risk both in livestock manure or soil. Specifically, in soil samples, the severity of ecological risks obtained a descending order as QNs > TCs > SAs, where six QNs (SAR, LOM, NOR, CIP, OFL, and MAR) and two TCs (TC and DOC) posed high risk, while all SAs revealed insignificant risk, illustrating the ecological risk of antibiotics in soil was in a quite severe situation and was mainly due to contamination of QNs, which had a strong sorption under suitable pH and cation exchange condition [51]. This result was similar as a previous survey in Jiangsu and Zhejiang provinces of China [46]. Additionally, the ecological risk from livestock manure exhibited a relatively higher extent compared to soil, where all QNs obtained medium or high risk among all species, all TCs became high-level risk in swine manure, and all SAs posed medium or high level in some samples, suggesting that antibiotics in livestock manure would probably increase the potential ecological risk to sensitive organisms in soil by the extensively utilization of livestock manure as fertilizer [52]. Antibiotics residues could decrease soil bacterial or fungal community diversity and change microbial community structure [33]; thus, the reuse of manure as organic fertilizers in farmland is more likely to be an essential pathway for antibiotic contamination, which could not be ignored.

Figure 5b–d show the ecological risk assessment of antibiotics to green algae (*Chlorella vulgaris* for all), water flea (*Ceriodaphnia dubia* for MAR and *Daphnia magna* for other antibiotics), and inflated duckweed (*Lemna gibba* for all) in water, respectively. All chosen species were sensitive to antibiotics in aquatic environment [53]. Obviously, for surface water, NOR displayed a medium-high and high risk to green algae and water fleas, respectively, and CIP displayed medium-high to both green algae and inflated duckweed in surface water. The ecological risk level associated with QNs were notably higher than SAs and TCs, consistent with prior research by Wei et al. [7] and Azanu et al. [54], indicating the detrimental impact on aquatic organisms. In addition, DOC also obtained a high-level risk to water fleas, which was primarily attributed to its lower risk thresholds (lower PNEC value). Moreover, for swine wastewater (liquid manure), the severity of ecological risks obtained a descending order as TCs > SAs > QNs, where three TCs (OTC, TC, and CTC) posed a high risk to green algae and inflated duckweed, as well as medium-high risk to water flea. A previous study also illustrated that TCs had higher ecological risk to aquatic organism [44]. However, the risk of DOC from swine wastewater showed insignificant level, much lower than that in surface water itself.

Meanwhile, as for other antibiotics, different ecological risks were obtained to different aquatic organism. For instance, SMX posed a heavy risk level to all organisms, which was much more serious to inflated duckweed, indicating that inflated duckweed was vulnerable to SMX exposure. ENR, NOR, CIP, SMX, and SMM posed medium-heavy lever to green algae in most samples, significantly higher than that to water fleas and inflated duckweed, suggesting green algae was generally more susceptible to antibiotics in water [53,55]. In summary, the ecological risk caused by antibiotics from swine wastewater deserves serious attention and recognition, since they would enter water body through runoff, leachate, or permeation, and it is crucial to scrutinize antibiotics linked to swine wastewater utilization as a fertilizer in the Sichuan Basin for risk management.

From the perspective of mixture effects of antibiotics on microorganisms, the harm of low antibiotics cannot be ignored due to the certain selective pressure brought by antibiotics residues. Except antagonistic or independent effects theoretically, additive or synergistic effects among antibiotics would probably strengthen the evolution and spread of ARGs [6], which is typically located on mobile genetic elements (MGEs), such as plasmids and transposons, consequently posing a significant threat to human health [56]. Although the mechanism of formation and transmission of ARGs is complex, and it is hard to clarify, the irreversible impairment of bacterial metabolism, and, consequently, the negative impacts to natural environments are obvious [57,58]. If physicochemical factors (pH, temperature, etc.) are suitable, the sensitivity and survivability of microorganisms are limited, the exposure time is long enough, and the ecological risk would become more severe. Hence, it is essential to give more attention to the antibiotic contamination from livestock excrement and their related risk to the environment and humans.

### 3.6. Limitations of the Study

Investigation into Sichuan Basin to understand the antibiotic occurrence from large-scale livestock farms is a very complicated and onerous work, requiring significant manpower investment, financial requirement, and time costs. Many factors interfere with the survey process, and limitation or uncertainty exist in this study. Firstly, determining what proportion of antibiotics in the external environment comes from livestock manure is still a challenge, because of the openness of the research system and the diversification of influencing factors. Secondly, the risk assessment in this study might be overestimated or underestimated without considering the mixture effects of various compounds. Thirdly, microbial toxicity tests cannot meet all the requirement when assessing the ecological risks; however, an integrated concept of the physiological potential of an inoculum (PPI) based on the biodegradation adaptation potential (BAP) and the chemical resistance potential (CRP), which includes both microbial toxicity tests and adequate biodegradation tests, would be helpful [57,59]. Therefore, it is hoped that future and more research can break through this problem.

## 4. Conclusions

This study provides a comprehensive investigation to illustrate the occurrence of 20 target antibiotics from large-scale livestock farms and environmental media (soil and aquatic water) in Sichuan Basin, China. Three categories of antibiotics were all detected with significantly varied concentrations among different livestock farms and different livestock species. QNs and TCs were ubiquitous in livestock excrement, where TCs obtained the highest average concentrations as 863 and 235 μg·kg^−1^ in swine farms, QNs obtained the highest average concentrations as 3155 and 76 μg·kg^−1^ in layer farms, and as 76 and 75 μg·kg^−1^ in cattle farms. Spatial distribution reveals that central, south, and southwest of Sichuan Basin displayed a higher contamination of antibiotics from livestock manure, which were considered as a possible source for their relatively higher abundance detected in soil according to source analysis, while other sources were responsible for the detection of antibiotics in surface water. The ecological risk assessment indicates that the resourceful utilization of livestock manure as fertilizer would probably elevate risk to a high level caused by antibiotic exposure to sensitive organisms living in soil and aquatic water, especially TCs and QNs from swine manure. However, the use of antibiotics in livestock farming is inevitable, and preventing and solving antibiotic contamination will require better management and further research.

## Figures and Tables

**Table 1:** Toxicity data of 20 antibiotics corresponding to sensitive species.

Group	Antibiotics	logK_ow_	*PNEC_water_* (μg·L^−1^) ^a^			*PNEC_soil_* (μg·kg^−1^) ^b^
			Green Algae ^c^	Water Flea ^d^	Inflated Duckweed ^e^	Soil
Tetracyclines (TCs)	Doxycycline (DOC)	−1.365	0.1	0.003	0.016	0.001
	Tetracycline (TC)	−1.392	0.05	0.072	0.055	0.005
	Oxytetracycline (OTC)	−2.867	0.066	4.028	0.075	0.005
	Chlortetracycline (CTC)	−0.684	0.05	1.868	0.033	0.004
Sulfonamides (SAs)	Sulfamethazine (SMZ)	0.757	5.804	3.3	0.473	0.163
	Sulfadiazine (SDZ)	−0.338	2.609	15.474	\	0.353
	Sulfathiazole (STZ)	0.715	\	4.757	\	1.569
	Sulfadimidine (SMD)	0.198	19.52	\	\	3.884
	Sulfamonomethoxine (SMM)	0.198	19.52	\	\	3.884
	Sulfachloropyridazine (SCP)	0.484	32.25	\	\	8.364
	Sulfamethoxazole (SMX)	0.484	0.5	0.207	0.003	0.001
	Sulfadimethoxine (SDM)	1.174	1.961	5.277	0.1	0.056
Quinolones (QNs)	Marbofloxacin (MAR)	−2.92	\	1.637	\	0.133
	Ofloxacin (OFL)	−0.2	0.081	1	0.039	0.006
	Ciprofloxacin (CIP)	−0.001	0.166	1	0.1	0.017
	Enoxacin (ENX)	0.81	58.7	\	\	21.501
	Norfloxacin (NOR)	−0.306	0.029	0.327	0.913	0.004
	Enrofloxacin (ENR)	0.701	0.405	3.263	\	0.132
	Lomefloxacin (LOM)	0.312	\	\	0.01	0.002
	Sarafloxacin (SAR)	0.846	\	1	\	0.381

^a.^ The value of *PNEC_water_* was determined by the distribution curves for the HC_5_. ^b.^ The value of *PNEC_soil_* was calculated by the minimum *PNEC_water_* of different species. ^c.^ The species name of green algae was *Chlorella vulgaris* for all antibiotics. ^d.^ The species names of water flea were *Ceriodaphnia dubia* for MAR and *Daphnia magna* for other antibiotics. ^e.^ The species name of inflated duckweed was *Lemna gibba* for all antibiotics.

**Table 2:** Occurrence and concentration of antibiotics in soil samples (n = 20).

Compounds	Mean (μg·kg^−1^)	Min (μg·kg^−1^)	1st Quarter (μg·kg^−1^)	Median (μg·kg^−1^)	3rd Quarter (μg·kg^−1^)	Max (μg·kg^−1^)	Detected No.	Frequency (%)
Chlortetracycline (CTC)	0.125	\	\	\	\	2.5	1	5
Tetracycline (TC)	1.82	\	\	2.65	2.9	4.2	12	60
Oxytetracycline (OTC)	0.17	\	\	\	\	3.4	1	5
Doxycycline (DOC)	0.755	\	\	\	\	4.1	4	20
**∑TCs**	**2.87**	**\**	**\**	**2.9**	**4.05**	**7.8**	**\**	**\**
Sulfamethazine (SMZ)	\	\	\	\	\	\	0	0
Sulfadiazine (SDZ)	\	\	\	\	\	\	0	0
Sulfathiazole (STZ)	\	\	\	\	\	\	0	0
Sulfadimidine (SMD)	\	\	\	\	\	\	0	0
Sulfamonomethoxine (SMM)	\	\	\	\	\	\	0	0
Sulfachloropyridazine (SCP)	\	\	\	\	\	\	0	0
Sulfamethoxazole (SMX)	\	\	\	\	\	\	0	0
Sulfadimethoxine (SDM)	\	\	\	\	\	\	0	0
**∑SAs**	**\**	**\**	**\**	**\**	**\**	**\**	**\**	**\**
Marbofloxacin (MAR)	2.225	\	2.1	2.25	2.75	3.6	18	90
Ofloxacin (OFL)	1.88	\	\	\	3.05	12.2	8	40
Ciprofloxacin (CIP)	4.645	4.1	4.35	4.55	4.75	5.9	20	100
Enoxacin (ENX)	3.685	0	3.65	3.8	4	4.5	19	95
Norfloxacin (NOR)	4.31	3.6	3.9	4.1	4.45	6.1	20	100
Enrofloxacin (ENR)	\	\	\	\	\	\	0	0
Lomefloxacin (LOM)	1.825	\	\	2.6	2.8	4	13	65
Sarafloxacin (SAR)	1.225	\	\	\	2.6	3.2	9	45
**∑QNs**	**19.795**	**13.9**	**17.4**	**19.55**	**21.65**	**29.8**	**\**	**\**

**Table 3:** Occurrence and concentration of antibiotics in surface water samples (n = 20).

Compounds	Mean (ng·L^−1^)	Min (ng·L^−1^)	1st Quarter (ng·L^−1^)	Median (ng·L^−1^)	3rd Quarter (ng·L^−1^)	Max (ng·L^−1^)	Detected No.	Frequency (%)
Chlortetracycline (CTC)	0.00016	/	/	/	0.00012	0.00123	5	25
Tetracycline (TC)	0.00018	/	/	/	0.00032	0.00081	6	30
Oxytetracycline (OTC)	0.00085	/	/	0.00015	0.00127	0.00536	11	55
Doxycycline (DOC)	0.0004	/	/	0.00039	0.00055	0.00194	13	65
**∑TCs**	**0.00159**	**/**	**0.00045**	**0.0012**	**0.00187**	**0.008**	**/**	**/**
Sulfamethazine (SMZ)	0.0096	0.00073	0.00283	0.00479	0.0133	0.0653	20	100
Sulfadiazine (SDZ)	0.00951	0.00013	0.00077	0.00247	0.00875	0.101	20	100
Sulfathiazole (STZ)	0.00005	/	/	/	/	0.00039	3	15
Sulfadimidine (SMD)	0.00006	/	/	/	/	0.00085	2	10
Sulfamonomethoxine (SMM)	/	/	/	/	/	/	0	0
Sulfachloropyridazine (SCP)	0.00352	/	0.00047	0.00134	0.00289	0.0182	18	90
Sulfamethoxazole (SMX)	0.00002	/	/	/	/	0.0004	1	5
Sulfadimethoxine (SDM)	/	/	/	/	/	/	0	0
**∑SAs**	**0.02276**	**0.00152**	**0.00561**	**0.01087**	**0.02588**	**0.1845**	**/**	**/**
Marbofloxacin (MAR)	/	/	/	/	/	/	0	0
Ofloxacin (OFL)	/	/	/	/	/	/	0	0
Ciprofloxacin (CIP)	0.02987	/	0.00411	0.0154	0.02435	0.189	19	95
Enoxacin (ENX)	0.03574	/	/	0.00925	0.0349	0.158	14	70
Norfloxacin (NOR)	0.03179	/	0.00741	0.0215	0.0453	0.112	19	95
Enrofloxacin (ENR)	0.0035	/	/	/	0.00471	0.0284	7	35
Lomefloxacin (LOM)	0.00232	/	/	/	0.00281	0.0193	7	35
Sarafloxacin (SAR)	0.00102	/	/	/	/	0.00737	3	15
**∑QNs**	**0.10424**	**0.00478**	**0.02497**	**0.04905**	**0.1079**	**0.5067**	**/**	**/**

## Data Availability

The data presented in this study are available in the article and in the Supplementary Materials.

## Supplementary Materials

The following supporting information can be downloaded at: https://www.mdpi.com/article/10.3390/toxics13030154/s1, Table S1: The breeding scale of livestock in Sichuan Basin, China (2022); Table S2: The names, abbreviations and physicochemical properties of the 20 antibiotics; Table S3: Optimization results of MS/MS for 20 antibiotics; Table S4: Standard curves and their correlation coefficients of 20 antibiotics; Table S5: Occurrence and concentration of antibiotics in different livestock feces samples; Table S6: Occurrence and concentration of antibiotics in different animal manure samples.
