# Supplementary material for: Overall Evaluation of Antibiotics Occurrence from Large-Scale Livestock Farms in Sichuan Basin, China: Spatial Distribution, Source Apportionment, and Risk Assessment
Source: Toxics. 2025 Feb 23;13(3):154. doi: 10.3390/toxics13030154 (PMC11945991; doi:10.3390/toxics13030154)
Supplement: Supplementary file 1 [file toxics-13-00154-s001.zip › toxics-3463624-supplementary.pdf]

toxics

MDPI

Supplementary Material

Overall Evaluation of Antibiotics Occurrence from Large-Scale Livestock Farms in Sichuan Basin, China: Spatial Distribution, Source Apportionment, and Risk Assessment

Changmiao Lai [1], Zhikai Wang [1], Teng Gu [1], Lei Jian [1], Xiaoxia Meng [1], Qingjie Meng [2] and Dongdong Gao [1,*]

1 Sichuan Academy of Eco-Environmental Sciences, Chengdu 610041, China; yiyi06072025@163.com (C.L.); kylewang0103@126.com (Z.W.); guteng0913@gmail.com (T.G.); jianlei327@hotmail.com (L.J.); scshky@yeah.net (X.M.)
2 Sichuan Province Ecological Environment Monitoring Station, Chengdu 610031, China; mengqingjie202501@163.com
* Correspondence: hydrogeochemistry@126.com; Tel.: +86-15982284521

Table S1. The breeding scale of livestock in Sichuan Basin, China (2022).

Municipal units	Livestock Number (Inventory)		Unit:10 000 heads	
	Swine	Cattle	Sheep	Layer
Chengdu	274.03	6.97	35.64	3118.35
Zigong	111.96	5.09	50.16	1891.00
Luzhou	259.15	19.16	39.10	2952.38
Deyang	160.12	12.90	19.95	3154.56
Mianyang	249.75	27.08	68.95	3780.58
Guangyuan	251.47	26.70	52.72	2830.61
Suining	219.45	8.24	27.46	1807.65
Neijiang	148.68	4.57	44.73	2003.87
Leshan	180.68	6.33	25.13	2543.11
Nanchong	403.90	31.43	158.75	4890.46
Meishan	146.53	5.92	34.41	1880.79
Yibin	327.69	32.52	37.02	3019.07
Guangan	242.31	8.37	17.64	2333.28
Dazhou	274.66	63.82	92.06	3918.57
Yaan	94.54	10.81	16.16	663.67
Bazhong	213.16	43.03	79.82	892.29
Ziyang	182.95	4.03	70.51	1738.53
**Sichuan Basin in all**	**3741.03**	**316.96**	**870.22**	**43418.77**
**Sichuan in all**	**4158.55**	**868.72**	**1529.85**	**45473.74**
**Proportion(%)**	**90.0**	**36.5**	**56.9**	**95.5**

Data sourced from 2022 statistics.

Table S2. The names, abbreviations and physicochemical properties of the 20 antibiotics.

Group	Compound	Acronym	CAS	Molecular Mass
Tetracyclines (TCs)	Chlortetracycline	CTC	57-62-5	478.88
	Tetracycline	TC	60-54-8	444.43
	Oxytetracycline	OTC	79-57-2	460.43
	Doxycycline	DOC	564-25-0	526.97
Sulfonamides (SAs)	Sulfamethazine	SMZ	57-68-1	278.33
	Sulfadiazine	SDZ	68-35-9	250.28
	Sulfathiazole	STZ	72-14-0	255.32
	Sulfadimidine	SMD	651-06-9	280.30
	Sulfamonomethoxine	SMM	1220-83-3	280.30
	Sulfachloropyridazine	SCP	80-32-0	284.72
	Sulfamethoxazole	SMX	723-46-6	253.28
	Sulfadimethoxine	SDM	122-11-2	310.33

Quinolones (QNs)	Marbofloxacin	MAR	115550-35-1	362.36
	Ofloxacin	OFL	82419-36-1	361.37
	Ciprofloxacin	CIP	85721-33-1	331.34
	Enoxacin	ENX	74011-58-8	320.32
	Norfloxacin	NOR	70458-96-7	319.33
	Enrofloxacin	ENR	93106-60-6	359.39
	Lomefloxacin	LOM	98079-51-7	351.35
	Sarafloxacin	SAR	91296-87	385.36

Table S3. Optimization results of MS/MS for 20 antibiotics.

Group	Compound	Acronym	Parention (m/z)	Daughterion I (m/z)	Daughterion II (m/z)	Mode	DP (V)		CE (V)	
							I	II	I	II
**Tetracyclines (TCs)**	**Chlortetracycline**	**CTC**	**479.2**	**444.1***	**462.1**	+	35.9	76.6	30.9	19.0
	Tetracycline	TC	445.2	428.1	410.1*	+	47.3	32.1	25.3	20.0
	Oxytetracycline	OTC	461.2	443.2	426.3*	+	39.3	43.0	22.1	28.0
	Doxycycline	DOC	445.3	466.0	154.0*	+	50.0	37.8	22.4	35.0
Sulfonamides (SAs)	Sulfamethazine	SMZ	279.1	124.1*	186.3	+	51.0	55.7	27.0	19.8
	Sulfadiazine	SDZ	251.1	156.1*	108.2	+	39.9	34.5	18.8	32.0
	Sulfathiazole	STZ	256.3	156.3*	108.3	+	40.0	45.9	20.2	28.4
	Sulfadimidine	SMD	281.1	156.2*	108.3	+	47.2	49.2	20.8	30.0
	Sulfamonomethoxine	SMM	281.1	156.2*	108.3	+	47.2	49.2	20.8	30.0
	Sulfachloropyridazine	SCP	285.0	156.1*	108.3	+	37.7	31.0	18.0	28.1
	Sulfamethoxazole	SMX	254.1	156.3*	108.1	+	35.8	32.8	17.0	33.7
	Sulfadimethoxine	SDM	311.1	156.2*	108.3	+	53.1	47.8	22.1	36.0
Quinolones (QNs)	Marbofloxacin	MAR	363.2	345.4	72.1*	+	55.0	39.3	22.1	46.3
	Ofloxacin	OFL	362.1	261.2*	318.2	+	46.1	44.9	32.8	33.0
	Ciprofloxacin	CIP	332.1	231.1	314.3*	+	41.3	52.8	46.2	27.7
	Enoxacin	ENX	321.1	232.0	303.3*	+	54.0	41.1	39.9	26.0
	Norfloxacin	NOR	320.2	276.3	302.3*	+	41.0	43.3	22.1	28.2
	Enrofloxacin	ENR	360.2	342.2*	316.4	+	49.9	45.9	30.1	33.0
	Lomefloxacin	LOM	352.2	265.3*	334.2	+	42.2	42.8	28.0	20.9
	Sarafloxacin	SAR	386.1	368.4*	299.2	+	41.0	47.0	33.0	34.2

* represents quantitative ion.

Table S4. Standard curves and their correlation coefficients of 20 antibiotics.

Group	Compound	Acronym	Internal indicator	Standard curves	Correlation coefficients
Tetracyclines (TCs)	Doxycycline	DOC	LIN-d_3	y=0.03509x-0.00032	0.9995
	Tetracycline	TC		y=0.08397x-0.00002	0.9999
	Oxytetracycline	OTC		y=0.05790x-0.00010	0.9998
	Chlortetracycline	CTC		y=0.03251x+0.00030	0.9997
Sulfonamides (SAs)	Sulfamethazine	SMZ	SUM-$^{13}C_6$	y=1.77981x+0.05621	0.9996
	Sulfadiazine	SDZ		y=0.35219x-0.00189	0.9998
	Sulfathiazole	STZ		y=0.15082x-0.00607	0.9995
	Sulfameter	SMD		y=0.30278x+0.01421	0.9997
	Sulfamonomethoxine	SMM		y=0.42064x+0.00714	0.9997
	Sulfachloropyridazine	SCP		y=0.36369x+0.00514	0.9998
	Sulfamethoxazole	SMX		y=0.45117x+0.00808	0.9999
	Sulfadimethoxine	SDM		y=1.08304x+0.00353	0.9999
Quinolones (QNs)	Marbofloxacin	MAR	CIP-d_8	y=3.63882x+0.19169	0.9998
	Ofloxacin	OFL		y=5.45155x+0.17536	0.9998
	Ciprofloxacin	CIP		y=5.20411x-0.14509	0.9997
	Enoxacin	ENX		y=11.9444x-0.27140	0.9999
	Norfloxacin	NOR		y=7.62925x-0.60100	0.9994
	Enrofloxacin	ENR		y=7.61838x+0.55542	0.9997
	Lomefloxacin	LOM		y=4.87774x+0.02187	0.9999
	Sarafloxacin	SAR		y=4.70247x+0.05061	0.9998

Table S5. Occurrence and concentration of antibiotics in different livestock feces samples.

Compounds	Swine (n=13)-Solid		Swine (n=6)-Liquid		Cattle (n=4)-Solid		Layer (n=5)-Solid		Sheep (n=3)-Solid	
	Range (μg·kg^{-1})	Frequency (%)	Range (ng·L^{-1})	Frequency (%)	Range (μg·kg^{-1})	Frequency (%)	Range (μg·kg^{-1})	Frequency (%)	Range (μg·kg^{-1})	Frequency (%)
CTC	15.8~4319	53.8	680~4457	66.7	Not detected	0	18.6	20.0	Not detected	0
TC	2~196	100	134~9743	66.7	2.8~8.3	75.0	4.0~19.0	80.0	2.2~16.7	100
OTC	3.2~1273	84.6	3380~69714	66.7	1.6	25.0	4.7~54.6	40.0	Not detected	0
DOC	1.6~177	84.6	Not detected	0	2.1~8.1	100	4.5~16.8	40.0	2.3	33.3
**∑TCs**	**6.1~4644**	\	**873~83914**	\	**3.3~16.4**	\	**8.5~90.4**	\	**4.5~16.7**	\
SMZ	2.9~37.6	38.5	135~511	66.7	6.1	25.0	3.7~5.7	80.0	3.9~6.7	66.7
SDZ	9.8~65.5	30.8	Not detected	0	4.5~5.4	50.0	5.8	20.0	Not detected	0
STZ	4.3~46.9	30.8	Not detected	0	17.6	25.0	4.9~7.8	40.0	5.2	33.3
SMD	2.6~5.0	38.5	Not detected	0	7.4	25.0	2.7~5.5	100	2.8~3.2	66.7
SMM	2.2~204	69.2	874	16.7	3.8	25.0	1.9	20.0	Not detected	0
SCP	14.9~166	23.0	Not detected	0	4.9	25.0	5.3~16.1	40.0	9.7	33.3
SMX	4.4	7.7	162~6940	66.7	3.5~5.4	50.0	3.0~3.1	40.0	Not detected	0
SDM	2.7	7.7	Not detected	0	2.3	25.0	Not detected	0	Not detected	0
**∑SAs**	**9.7~241.6**	\	**162~7949**	\	**4.5~35.3**	\	**9.8~23.4**	\	**11.9~19.6**	\
MAR	1.9~24.2	100	Not detected	0	2.0~8.5	100	2.5~11.2	100	2.0~8.2	100
OFL	2.9~33.4	53.8	Not detected	0	3.0	25.0	3.4~5.4	80.0	3.3~3.5	66.7
CIP	4.0~148	100	140~690	50.0	5.3~30.7	100	9.7~835	100	4.6~100	100
ENX	3.6~20.4	100	Not detected	0	3.7~16.1	100	14.4~23.5	80.0	3.6~24.2	100
NOR	3.4~24.3	100	77~742	83.3	3.5~15.2	100	6.8~23.8	80.0	3.7~18.7	100
ENR	4.5~3759	100	55~1031	88.3	4.9~8.7	50.0	4~14520	80.0	5.8~6.0	66.7
LOM	2.4~24.7	46.1	Not detected	0	2.7~9.3	50.0	9.7~10.6	60.0	2.4~7.8	100
SAR	3.4~17.4	61.5	Not detected	0	2.5~8.4	75.0	7.6~26.3	80.0	2.5~8.9	100
**∑QNs**	**37.1~4013.7**	\	**271~2289**	\	**16.5~99.9**	\	**19~15424.3**	\	**18.8~166.5**	\

Table S6. Occurrence and concentration of antibiotics in different animal manure samples.

Compounds	Swine (n=18)-Solid		Swine (n=27)-Liquid		Cattle (n=4)-Solid		Layer (n=5)-Solid		Sheep (n=1)-Solid	
	**Range ($\mu g \cdot kg^{-1}$)**	**Frequency (%)**	**Range ($ng \cdot L^{-1}$)**	**Frequency (%)**	**Range ($\mu g \cdot kg^{-1}$)**	**Frequency (%)**	**Range ($\mu g \cdot kg^{-1}$)**	**Frequency (%)**	**Range ($\mu g \cdot kg^{-1}$)**	**Frequency (%)**
CTC	2.7~23.1	38.9	64~214000	88.9	Not detected	0	1.5	20.0	13.6	100
TC	3.0~24.2	88.9	66~3160	74.1	2.1~41.4	100	3.4~35.0	80.0	11	100
OTC	16.3~2266	55.6	22~54000	74.1	Not detected	0	Not detected	0	Not detected	0
DOC	1.9~29.7	83.3	Not detected	0	7.1	25.0	2.0~17.0	60.0	13.9	100
**∑TCs**	**1.9~2284.1**	\	**45~214274**	\	**2.1~41.4**	\	**4.8~36.5**	\	**38.5**	\
SMZ	3.2~25.6	38.9	14~243	66.7	3.3~5.1	50.0	3.7~4.6	60.0	Not detected	0
SDZ	4.9~43.9	38.9	Not detected	0	Not detected	0	6.5~129	40.0	Not detected	0
STZ	4.1	5.6	Not detected	0	7.2~9.1	50.0	4.0~11.0	60.0	Not detected	0
SMD	2.7~5.4	55.6	Not detected	0	2.8~7.1	75.0	2.8~4.8	60.0	Not detected	0
SMM	3.1~199	38.9	9~38200	66.7	Not detected	0	8.5	20.0	Not detected	0
SCP	8~568	27.8	Not detected	0	4.6	25.0	Not detected	0	Not detected	0
SMX	3.4~4.5	11.1	29~92200	81.5	Not detected	0	Not detected	0	Not detected	0
SDM	2.3~3.0	11.1	Not detected	0	4.4	25.0	Not detected	0	Not detected	0
**∑SAs**	**3.0~620.2**	\	**42~130545**	\	**3.1~30.3**	\	**13.1~137.5**	\	**Not detected**	\
MAR	2.0~12.6	88.9	Not detected	0	2.3~9.6	50.0	2.2~9.2	80.0	Not detected	0
OFL	3.2~9.6	44.4	Not detected	0	2.7~10.2	100	3.9~6.2	60.0	3.2	100
CIP	4.4~92.6	100	5~399	100	3.9~26.8	100	5.0~26.2	100	18.1	100
ENX	3.6~19.9	94.4	Not detected	0	3.9~31.6	100	3.7~19.1	100	18.2	100
NOR	3.9~38.6	100	2~432	100	4.4~31.7	100	4.7~31.5	80.0	15.2	100
ENR	7.5~907	88.9	11~740	96.3	4.6~5.1	50.0	3.4~26.0	80.0	7.9	100
LOM	2.7~10.7	55.6	Not detected	0	7.7~12.0	75.0	3.4~9.2	60.0	7.8	100
SAR	2.5~15.2	72.2	Not detected	0	2.9~14.2	100	3.6~14.1	100	8.1	100
**∑QNs**	**22.3~1018.8**	\	**7~1571**	\	**20.1~131.1**	\	**18.4~124.4**	\	**78.5**	\
